# Use of Probiotics and Oral Health

**DOI:** 10.1007/s40496-017-0159-6

**Published:** 2017-10-19

**Authors:** Robert P. Allaker, Abish S. Stephen

**Affiliations:** Institute of Dentistry, Queen Mary University of London, London, UK

**Keywords:** Probiotics, Periodontal diseases, Caries, Microbiome, Nitric oxide, Oral malodour

## Abstract

**Purpose of Review:**

The purpose of this study is to critically assess recent studies concerning the use of probiotics to control periodontal diseases, dental caries and halitosis (oral malodour).

**Recent Findings:**

Clinical studies have shown that probiotics when allied to conventional periodontal treatment can ameliorate microbial dysbiosis and produce significant improvement in clinical indicators of disease. However, this effect is often not maintained by the host after the end of probiotic use. Current probiotics also show limited effects in treating caries and halitosis. Novel approaches based up on replacement therapy and using highly abundant health-associated oral species, including nitrate-reducing bacteria, have been proposed to improve persistence of probiotic strains and maintain oral health benefits.

**Summary:**

Probiotics have potential in the management of multifactorial diseases such as the periodontal diseases and caries, by more effectively addressing the host-microbial interface to restore homeostasis that may not be achieved with conventional treatments.

## General Introduction

The prevention of the plaque-related diseases, dental caries and the periodontal diseases, normally involves the non-specific control of dental plaque as this is the initiating factor. This is carried out to maintain levels of dental plaque compatible with health and thus prevent the breakdown of microbial homeostasis (dysbiosis) concomitant with disease risk. However, the individual response of the host and other confounding factors can influence disease initiation and progression. Antimicrobial and more general antiplaque compounds in oral care products represent a significant complement to mechanical plaque control. Such approaches should preferably reduce oral biofilm formation without affecting the biological equilibrium within the oral cavity, which is inhabited by approximately 1000 different species of bacteria at 10^8^–10^9^ bacteria per mL saliva or mg dental plaque [1]. However, with ever increasing resistance to antibiotics and a desire from the general public for more ‘natural’ therapies, there is a need to minimise antibiotic use and develop novel treatments for oral diseases that do not involve conventional antimicrobial agents [2]. Preventive approaches based upon the restoration of the microbial ecological balance, rather than elimination of the disease associated species, have been proposed [3]. These include the use of prebiotics to promote health-associated bacterial growth or the use of probiotic bacteria with associated benefits. As regards the possible side effects of oral probiotics, these are likely to be mild in healthy individuals and possibly digestive as observed with those used to control intestinal disease [3]. In theory, they could cause systemic infections that require treatment with antimicrobial agents, particularly in individuals with underlying health conditions.

## Probiotics—Introduction

Probiotics, by definition are viable microorganisms which, when administered in adequate amounts, provide a health benefit to the host. This approach has successfully been used to control intestinal diseases and appears to act through colonisation resistance and/or modulation of the immune system [2]. Likewise, studies are now suggesting that probiotics have the potential to modify the oral microbiota and are being investigated to prevent or treat diseases of the oral cavity, such as dental caries and the periodontal diseases, which are associated with a shift in the microbial composition and activity of the biofilm, and the resulting reaction of the host [2]. Strains belonging to the *Lactobacillus*, *Streptococcus* and *Bifidobacterium* genera are most commonly investigated as regards probiotics. Species within these taxa are members of the normal microbiota found within the gastrointestinal tract with some species preferentially colonising the oral cavity. With regards to periodontitis, there are health- and disease-associated *Streptococcus* spp., while species from the aforementioned three genera have all been associated with dental caries [3]. Therefore, when selecting suitable probiotic species, the normal oral habitat and association with health should be considered.

Experimental studies and clinical trials have demonstrated that certain gastrointestinal bacteria, including *Lactobacillus* and *Bifidobacterium* spp., have the potential to control the growth of oral microorganisms, including the cariogenic streptococci [4]. Within the oral cavity, mechanisms of probiotic action can possibly be suggested from previous gastrointestinal studies, whereby the introduction of microorganisms as a therapeutic tool for the control of oral and dental disease could act as follows [4]:

A. Direct interactions within dental plaque (colonisation resistance). This mechanism could possibly include the disruption of plaque biofilm formation through competition for binding sites on host tissues and other bacteria, and through competition for nutrients. The production of antimicrobial compounds by probiotic species that inhibit other oral bacteria may also be a significant mechanism. It is known that lactic acid bacteria produce a range of antimicrobial agents including organic acids, hydrogen peroxide, peptides, bacteriocins and anti-adhesion molecules [4].
B. Indirect probiotic actions within the oral cavity, including the modulation of both innate and adaptive immune function. Within this context, it is possible that lactic acid bacteria can interact with immunocompetent cells, such as macrophages and T-cells, leading to an alteration in the production of cytokines and subsequent effects on overall immunity [4]. For example, lactobacilli are able to elicit a transient reduction in IL-8 secretion in the gingival crevicular fluid of subjects with mild gingival inflammation. Beyond the modulation of immune responses, some probiotic species are able to enhance mucin production and barrier function, upregulate host defence peptides, promote angiogenesis and wound healing [5].

## Periodontal Diseases

Periodontal diseases (periodontitis and gingivitis) are a group of inflammatory pathologies of the periodontium that lead to loss of teeth principally due to dysregulated, immune-mediated destruction of the periodontal ligaments and tooth supporting structures [6, 7]. A dysbiotic oral microbiota is associated with periodontitis and in the most common forms of this disease namely, chronic and aggressive periodontitis is thought to play an active role in the pathogenesis by promoting chronic dysregulated inflammation which in turn sustains the dysbiotic microbial ecology [8, 9].

### Probiotics as an Adjunct for Clinical Periodontal Treatment

A probiotic that could alter the oral microbial ecology may be a useful tool in the clinical management of periodontitis, with the potential to offer two-fold benefits [10]. Firstly, to combat dysbiosis by competitive inhibition of periodontal pathogens, and thereby reducing the overall immunogenicity of the oral microbiota. Secondly, to modulate active disease-associated immune/inflammatory pathways to reduce the destructive inflammation of periodontitis, and lead to immune homeostasis that could be maintained by the host in the long term.

Clinical studies in humans that have explored treatment of the periodontal diseases using probiotic therapy unaided by clinical treatment measures, have reported modest overall benefits such as reduction of gingival bleeding and probing depth [11]. However, studies that involved probiotics as an adjunct to clinical periodontal treatment report a more marked improvement in the clinical status of patients compared to clinical treatment alone (Table 1). This could be an important avenue for probiotics in lieu of antibiotics in periodontal treatment to help reduce the overall burden of antibiotic resistance [28, 29]. Despite existing heterogeneity in the clinical studies that investigated use of probiotics in the management of gingivitis or periodontitis, meta-analyses in the literature have found some overall support for the use of probiotics [30, 31•]. However, there are several aspects to probiotic therapy that need to be better understood before routine use in managing gingivitis and periodontitis can be recommended [32]. These include: (i) length and mode of treatment to prevent the periodontal microbiota from reverting to a dysbiotic ecology at termination of treatment (ii) characterising the possible in vivo cariogenic effects of the probiotic strains which may manifest during the treatment period (iii) elucidating the possible systemic risks of administering probiotic strains to individuals with systemic pathologies that involve mild to moderate immune suppression.

**Table 1:** Summary of the clinical studies that evaluated various probiotic strains for treatment of chronic periodontitis or gingivitis

Reference	Probiotic strain(s) (mode of delivery)	Study population	Design	Probiotic treatment	Monitoring	Clinical measurements			
						Plaque indices	Bleeding indices	Probing depth	Clinical Attachment Loss
[12]	*Lactobacillus plantarum Lactobacillus brevis Pediococcus acidilactici* (tablet)	Gingivitis minor attachment loss < 2 mm	Parallel-group	Two tablets/day for 6 weeks	Baseline and week 6	ns	↓ AngBS ↓ GI	–	–
[13]	*Lactobacillus reuteri* DSM 17938 and ATCC PTA 5289 (lozenge)	Gingivitis pregnant women in 3rd trimester	Parallel-group	Two lozenges/day for 7 weeks	Baseline and week 7	↓ PI	↓ GI	–	–
[14]	*Lactobacillus salivarius Lactobacillus reuteri* (capsule/direct)	Chronic periodontitis CAL ≥4 mm ≥ 4 sites with PD ≥5 mm	Parallel-group SRP+adjunct	Twice/day for 14 days rinse with capsule direct subgingival delivery at baseline, weeks 1, 2 and 4.	Baseline, months 1 and 3	↓ PI up to 3 months	↓ GI and BI up to 3 months	↓ ns	↓ ns
[15]	*Lactobacillus rhamnosus* (sachet)	Chronic periodontitis ≥ 5 teeth with PD ≥ 5 mm and CAL ≥3 mm	Parallel-group SRP+adjunct	1/day drink of sachet contents for 3 months	baseline, 6 weeks, and months 3, 6, 9 and 12	↓ ns at 12 months	ns	↓ ≥ 6 mm sites	↓ ns at 12 months
[16]	*Lactobacillus plantarum* L-137 (heat killed; capsule)	‘Chronic periodontitis’ patients undergoing therapy ≥1 site PD ≥4 mm	Parallel-group SRP+adjunct	1/day for 12 weeks	Baseline and weeks 4, 8 and 12	ns (PI)	↑ BOP ns up to 12 weeks	↓ sites ≥ 4 mm ns	–
[17]	*Bacillus subtilis Bacillus megaterium Bacillus pumulus* (toothpaste, toothbrush cleaner, mouth rinse)	Gingivitis	Parallel-group	2/day brushing with probiotic toothpaste and 1/day mouth rinse for 8 weeks (toothbrush stored in cleaner till next use)	Baseline and week 8	ns (PI)	ns (GI)	–	–
[18]	*Streptococcus oralis* KJ3 *Streptococcus uberis* KJ2 *Streptococcus rattus* JH145 (tablet)	Chronic periodontitis (severe)	Parallel-group SRP+adjunct	2/day dissolving tablet on tongue 12 weeks	Baseline and weeks 4, 8, 12 and 24	PI ↓ up to 24 weeks	↓ GI and ↓ BOP ns up to 24 weeks	ns up to 24 weeks	ns up to 24 weeks
[19]	*Lactobacillus reuteri* (lozenge)	Chronic periodontitis radiographic evidence of horizontal bone loss; ≥ 2 teeth per quadrant at 5–7 mm PD; ≥ 2 GI per quadrant	Parallel-group SRP+adjunct	2/day for 3 weeks.	Baseline and days 21, 90, 180 and 360	PI ↓ up to 360 days	↓ GI and BOP up to 360 days	↓ ≥ 5 mm sites up to 360 days	↓ up to 360 days
[20]	*Lactobacillus brevis* CD2 (lozenge)	Experimental gingivitis	Parallel-group abstained from oral hygiene after cleaning period for 2 weeks	3/day for 2 weeks	Baseline, 3, 7, 10 and 14 days	ns (PI)	↓ BOP at Day 10 only.	–	–
[21]	*Lactobacillus reuteri* ATCC PTA 5289 (tablet)	Moderate chronic periodontitis CAL > 5 mm and PD > 4 mm in ≥ 2 adjacent teeth	parallel-group SRP+adjunct	2/day (time period not clear)	Baseline, end time point 2 weeks after probiotics terminated	ns (PI)	↓ SBI	↓ mean PD	↓
[22]	*Lactobacillus reuteri* ATCC 55730 and ATCC PTA 5289 (lozenge)	Experimental gingivitis (female participants only)	Cross over professional tooth cleaning during a washout period of 2 weeks.	2/day for 3 weeks	Baseline and 21 days	ns (PI)	ns (GI and BOP)	–	–
[23]	*Lactobacillus reuteri* DSM 17938 and ATCC PTA 5289 (lozenge)	Chronic periodontitis (moderate-severe)	Parallel-group Full mouth disinfection and SRP+adjunct	2/day for 12 weeks	Baseline and week 3, 6, 9 and 12	↓ ns at 3, 9 and 12 weeks ↓ at 6 weeks	↓ ns at 3, 6 and 9 weeks ↓ at 12 weeks	↓ ns mean PD ↓ ≥ 5 mm PD at 12 weeks	↓ ns
[24]	*Lactobacillus reuteri* ATCC 55730 and ATCC PTA 5289 (tablet)	Chronic periodontitis	Parallel-group no professional treatment	1/day for 30 days	Baseline and day 30	↓ at day 30	↓ BOP at day 30	↓ 4–5 mm PD ↓ ≥ 6 mm PD at day 30.	–
[25]	*Lactobacillus reuteri* DSM 17938 and ATCC PTA 5289 (tablet)	Gingivitis > 1 gingival index and no CAL	Parallel-group teeth polished at baseline	1/day for 28 days.	Baseline, week 4 and 8	ns (mean PI)	ns (mean GI)	–	–
[26]	*Lactobacillus casei ‘*Shirota’ (milk)	Experimental gingivitis	Parallel-group scaling and polishing 2 weeks before baseline	1/day drink (65 mL) for 28 days	Baseline and day 1, 3, 5, 7 and 14	Mean PI at baseline for two groups were different.	↓ BOP up to 14 days GI values were significantly different at baseline	–	–
[27]	*Lactobacillus reuteri* DSM 17938 and ATCC PTA 5289 (lozenge)	Chronic periodontitis PD 5–7 mm and radiographic bone loss evidence	Split-mouth SRP+adjunct	2/day for 3 weeks; 3 week gap after SRP at baseline	Baseline, week 3 and 6	↓ PI score at week 6	↓ GI score at week 6	↓ mean PD at week 6	↓ at week 6

*PI* plaque index, *GI* gingival index, *GBI* gingival bleeding index, *AngBS* angulated bleeding score, *BI* bleeding index, *SBI* sulcus bleeding index, *BOP* bleeding on probing (% of sites), *PD* probing depth, *CAL* clinical attachment loss, *SRP* scaling and root planing, *ns* not significant

↓decrease

↑increase

### Probiotics in Remodelling the Periodontal Ecosystem

The periodontium provides several unique niches for microbial colonisation, with the subgingival niche being the most studied in relation to the periodontal diseases [33, 34]. Most studies investigating the role of probiotics in modifying the microbial ecology in the periodontium in vivo have endeavoured to measure specific microbial species considered to be ‘periodontopathogens’ or key microorganisms in oral biofilm development. In a study using lozenges containing *Lactobacillus reuteri* strains as an adjunct to scaling and root planing treatment of chronic periodontitis, significant reductions in the abundance of *Porphyromonas gingivalis* in saliva, subgingival and supragingival plaque were reported in the treatment group. However, no significant reductions in the overall plaque scores compared to the group who underwent clinical treatment and consumed placebo lozenges were demonstrated [23]*.* Streptococcal probiotic strains evaluated as adjuncts to clinical treatment of chronic periodontitis showed significant reduction in plaque scores, in addition to reductions of *Tannerella forsythia* and *Prevotella intermedia* in supragingival plaque and saliva, respectively [18]. Microbiological culture of subgingival plaque in a split-mouth study with *L. reuteri* as an adjunct to conventional treatment in chronic periodontitis patients has also shown reductions in *Aggregatibacter actinomycetemcomitans, P. gingivalis* and *P. intermedia* [27]*.* Whilst the differences in dental plaque scores between these studies may relate to the probiotic strain used and differences in treatment modality, these data suggest that probiotic strains could alter the community structure of the supragingival and subgingival plaque in chronic periodontitis, either directly by inhibiting disease-associated taxa such as *Fusobacterium nucleatum* and *Prevotella intermedia* or indirectly by inhibiting the keystone pathogen *P. gingivalis*.

Probiotic treatment of gingivitis as an adjunct to professional mechanical plaque removal has also shown significant reductions in the major periodontopathogens namely, *P. gingivalis*, *A. actinomycetemcomitans* and *T. forsythia* in the subgingival plaque compared to placebo [12]. While treatment of gingivitis with *L. reuteri* lozenges alone has been reported to significantly reduce *P. gingivalis* and *A. actinomycetemcomitans* abundance in the subgingival plaque, *P. gingivalis* was reported to recover and show an increase in prevalence in the subgingival plaque within 4 weeks after the end of treatment [25]. In a study that investigated twice per day probiotic *L. reuteri* consumption for 3 weeks as an adjunct to clinical treatment of chronic periodontitis, a significant reduction in the proportion of obligate anaerobes in the subgingival plaque was observed up to 21 weeks after the end of the probiotic course compared to placebo, with a return to baseline levels at the 1 year follow-up [19]. These observations suggest that while currently available probiotics could alter the periodontal microbial ecology to resemble a more health-associated ecology during treatment of gingivitis or chronic periodontitis, this effect is not maintained by the host in the long term. It has been suggested that this could be due to the low persistence of these probiotic strains in the oral cavity, as the majority used are not oral isolates, and those currently in use as probiotics are rare taxa in themselves and thus more susceptible to fluctuations in the oral environment [35]. Further, the lack of more comprehensive ecological surveys using established high throughput 16S rDNA sequencing platforms in the literature limits the current understanding into the nature of community-wide changes exerted by the probiotic strains in the periodontal niches during treatment.

### Probiotics in the Maintenance of Periodontal Health

Ecological surveys reported in studies that investigated probiotics for preventative oral care have provided some insights into the changes occurring in the oral microbiome of healthy individuals consuming probiotic products. A course of *Lactobacillus rhamnosus* GG and *Bifidobacterium animalis* ssp. *lactis* containing lozenges taken by healthy individuals were reported to show no significant changes in the salivary ecology compared to baseline as profiled by the human oral microbe identification microarray, but did allow an improvement in gingival health [36]. A study that employed next generation sequencing of salivary microbiota in healthy individuals consuming a transconjugant *Streptococcus salivarius* M18 also reported no significant changes in the overall ecology, with the probiotic streptococcus forming a large proportion of the total indigenous *S. salivarius* population in some individuals [37•].

However, significant health-associated changes in the supragingival plaque microbiota were reported in a study that employed *L. reuteri* as a prophylactic probiotic, suggesting niche-specific ecological changes, in addition to effects specific to the probiotic strain used [38•]. After a 12-week course of the probiotic, the supragingival microbiota was reported to be associated with an increased relative abundance of *Neisseria subflava*, *Campylobacter consisus*, *Granulicatella adicaens*, *Bergeyella* sp. HOT322, *Streptococcus oralis* and other health-associated oral taxa including nitrate-reducing species*.* The baseline samples showed an abundance of disease-associated taxa including *Streptococcus mutans*, *Fusobacterium periodonticum*, *F. nucleatum* ssp. *vincentii*, *S. anginosus*, *Eikenella* spp. and *Neisseria mucosa*. However, at the 1-month follow-up after termination of probiotic use, the community structure was found to revert to baseline, with a significant reduction in the prevalence of *L. reuteri* in the saliva.

### Immunomodulatory Activities of Probiotics in Disease and Health

Anti-inflammatory activity is a key effect of probiotics observed in vitro and as demonstrated in animal model studies, further supported by human studies in non-oral contexts [39, 40]. The systemic immunomodulatory effects of gut based probiotics may also have a protective effect in relation to periodontitis as demonstrated in a mouse model [41, 42]. In addition, it has been shown that probiotics can have a positive effect on oral health by means of reduced osteoclastic activity in a murine orthodontic tooth movement model [43].

In human experimental gingivitis studies with *L. reuteri* or *L. brevis* lozenge use, no marked differences in the gingival crevicular fluid (GCF) levels of cytokines such as TNF-alpha, IL-1 beta, CXCL8, CXCL10, CCL4, MMP-8 and prostaglandin E2 concentrations were reported compared to placebo [20•, 22]. While elevated IL-6 and lowered IL-18 concentrations were reported in these studies, no significant changes in the clinical parameters assessed in the individuals were found [22]. However, probiotic *L. reuteri* as an adjunct for clinical treatment of chronic periodontitis was reported to yield more marked anti-inflammatory effects in showing a reduction in TNF-alpha, IL-1 beta and IL-17 concentrations in the GCF of patients, allied to an improvement in clinical aspects of disease such as gingival bleeding index and probing depth [21]. There is limited evidence that some of these effects could be maintained in the medium term, as another study that followed chronic periodontitis patients for up to a year after a 3-week course of *L. reuteri* lozenges after scaling and root planning procedures found a reduction in GCF matrix metalloproteinase-8 (MMP-8) and an increase of tissue inhibitor of matrix metalloproteinase-1 (TIMP-1) up to 180 days [44]. These studies suggest that immune modulation is detectable within GCF when probiotics are used in conjunction with professional mechanical plaque removal and a sustained oral hygiene regimen, and this is exhibited along with an improvement in clinical oral health parameters.

Animal studies have shown a more pronounced activity for probiotics; however, these studies treat disease without mechanical dental plaque removal interventions. It has been shown that *Bifidobacterium animalis* ssp. *lactis* could confer protection from bone loss when administered subgingivally to ligature-induced periodontitis sites in a murine model [45]. Other studies have also shown protective effects against bone loss with *Bacillus subtilis*, *Bacillus licheniformis* and *L. brevis* CD2 in murine periodontitis models [46–48]. Recent in vitro studies continue to elucidate the mechanisms of probiotic immune modulation, showing that probiotic lactobacilli could abolish CXCL8 attenuation by *P. gingivalis*, and promote Th1 and Th17 responses [49, 50]. An 8-species probiotic mixture containing lactobacilli, bifidobacteria and streptococcus has been shown to polarise human macrophages towards the M1-phenotype [51].

## Halitosis (Oral Malodour)

Intra-oral halitosis is a common condition, and is known to be associated with periodontitis with the putrefactive activity of the tongue microbiota playing a major role in producing volatile malodorous compounds in both pathological (disease-associated) and physiological (transient non-disease associated) halitosis [52, 53]. Due to this distinction in the aetiology of intra-oral halitosis, probiotics that could help maintain periodontal health could also serve to combat pathological halitosis, while helping to maintain a healthy tongue ecology as, the more difficult to reach areas of the tongue for oral hygiene such as the dorsal posterior surface to the circumvallate papillae are known to harbour a high abundance of anaerobic gram-negative bacterial species associated with malodour [54]. However, it is known that the tongue is a more distinct niche than the periodontal niches in terms of species that normally colonise suggesting the requirement of niche-specific adaptations, and probiotic strains targeted to colonise the periodontal niches may not readily colonise the tongue to exert health-promoting effects [55, 56].

One of the earliest probiotic strains proposed to target oral malodour and fulfilling the above criteria was the bacteriocin-producing strain *S. salivarius* K12, which reduced breath volatile sulfur compound (VSCs) concentrations in individuals who consumed the probiotic lozenges after pre-treatment with a chlorhexidine rinse [57–60]. This strain has also been shown to inhibit in vitro growth of some oral malodour associated bacterial species such as *Solobacterium moorei*, *Parvimonas micra* and *Eubacterium sulci* [60]*.* Studies using *Lactobacillus salivarus* WB21 for a short course by individuals with oral malodour found that periodontal health improved in addition to a reduction in breath VSCs [61, 62]. However, whilst this study found a reduction in salivary abundance of the VSC producing species *F. nucleatum*, no significant differences in the organoleptic scores between treatment and placebo were found. Favourable anti-VSC activities have been demonstrated with in vitro evaluations using *Weisella cibaria*, *Enterococcus faecium* and *Streptococcus thermophilus*, although these are non-oral strains [63–65]. Lactobacilli that have shown positive effects with regards to treating periodontitis and promoting oral health have been evaluated in human clinical studies to reduce oral malodour. It has been shown that reuterin-like compounds could suppress VSC production by periodontopathic bacteria such as *F. nucleatum* and *P. gingivalis*, in addition to downregulation of the enzyme (methionine γ lyase) responsible for production of the VSC methanethiol in the latter [66]. However, *L. reuteri* consumption was not found to reduce organoleptic scores, and a cysteine rinse evaluation did not produce a significant difference to placebo [67]. Individuals with persistent oral malodour treated with *L. brevis* CD2 lozenges failed to show an improvement in organoleptic scores or breath VSC concentrations [68]. Consumption of *Lactobacillus casei* Shirota milk also did not show significant changes in the breath VSC concentration or organoleptic scores, despite presence of the probiotic strain in the tongue plaque during treatment [69].

These studies highlight that current probiotics show very limited ability to control oral malodour in vivo. There is a need to screen available strains for adherence to the tongue and to demonstrate tongue colonisation in clinical studies [70]. It may also be useful to show effects of probiotic strains targeted to the periodontal niches, on the tongue ecology. Further, more abundant tongue-associated indigenous oral strains could be screened for probiotic activity to help with colonisation and maintenance of healthy tongue ecology. Conversely, understanding the persistence mechanisms of the more disease-associated oral microbial species could also help in construction of novel low-abundance probiotic strains with enhanced persistence.

## Nitrate-Reducing Bacteria as Probiotic Agents

Nitric oxide (NO) is a labile and highly reactive gas which is known to be generated endogenously through the activity of NO synthases from mammalian cells and contributes to host defence against a number of pathogenic microorganisms [71]. The generation of NO, through the action of microbial nitrate reductases on salivary nitrate, has also been recognised as a significant source of this molecule [72•]. Nitrate absorbed from ingested dietary sources, especially green vegetables, is actively concentrated by the salivary glands so that concentrations in the saliva are approximately 10 times those found in plasma [73]. Nitrate is then rapidly converted to nitrite in the mouth by bacteria, through the activity of nitrate reductase enzymes. It has been shown that the bacteria responsible for nitrate reduction reside within the crypts of the tongue, where they are maintained in an anaerobic environment and reduce nitrate to nitrite during respiration [74]. The function of the salivary concentration of nitrate and reduction to nitrite is not fully established but it has been shown that the high concentrations of nitrite formed in saliva will, when acidified in the stomach, produce nitrous acid and NO in sufficient concentrations to kill *Escherichia coli* and other enteric pathogens [75]. Additionally salivary nitrite will encounter the acid environment around the teeth provided by acidogenic bacteria such as *Lactobacillus* spp. and *Streptococcus mutans*. In vitro studies have shown that acidified nitrite will significantly decrease the growth and survival of these bacteria [76]. It is thus hypothesised that increasing nitrate intake may be important in suppressing the growth of acid-forming bacteria and thereby protect the teeth against caries.

A relationship between dental caries and levels of nitrate and microbial nitrate reductase activity in the saliva of children has been established. Compared with control subjects, a reduction in caries experience was found in patients with high salivary nitrate and high nitrate-reducing ability [77]. Production of nitrite from salivary nitrate by commensal nitrate-reducing bacteria (NRB) may thus limit the growth of cariogenic bacteria as result of the production of antimicrobial oxides of nitrogen, including NO. This lends support to the hypothesis that a high nitrate-reducing flora may also be of benefit in the protection against dental caries. In recent oral microbiome studies based upon bacterial 16S rDNA sequencing using the HOMINGS methodology and Minimum Entropy Decomposition data analysis, the stability of the tongue microflora in health, particularly the NRB including *Rothia* spp. was shown to be associated with oral health [78].

The identification of NRB as commensal inhabitants may now provide the basis for probiotic therapy in mammals susceptible to oral infections. In man, the new-born infant is at first edentulous and has a microbial flora characteristic of this condition. Studies have shown that the frequency of isolation of NRB significantly increases after the teeth begin to erupt at about the age of 6 months [Allaker; unpublished observations]. Thus, probiotic therapy could be accomplished by the introduction of NRB into neonates, which have yet to acquire these bacteria or adults after the use of broad-spectrum antibiotics to firstly reduce tongue populations of bacteria. Bacterial metabolism of nitrate to nitrite within the oral cavity and the subsequent formation of biologically active nitrogen oxides are unlikely to have wider undesirable effects; however, disruption of the intestinal microflora remains a possibility.

It is well established that salivary glands may respond to periodontitis through the enhancement of the protective effects of saliva [79•]. An increase in nitrate secretion and subsequent increase in salivary nitrite through the activity of NRB has been found to be higher in subjects with periodontitis and thus may contribute to this protection in response to the inflammatory process [79•]. NO levels in saliva and gingival crevicular fluid have been found to be higher in patients with aggressive periodontitis as compared to gingivitis [80]. This could arise both through an increase in salivary nitrate and subsequent microbial reduction and also through the activity of host NO synthases where the upregulation of enzyme activity in response to periodontal bacteria has been shown [81]. Therefore the use of probiotics to enhance oral NO production through both nitrate reduction and upregulation of synthase activity may be beneficial in the control of bacteria associated with dental caries and periodontitis. However, the role of NO is both positive as an antimicrobial agent and negative as regards its inflammatory effects if present in high enough concentrations. Indeed, the probiotic strain *Lactobacillus brevis* CD2 has been shown to delay the development of gingivitis in a clinical model by the downregulation of the inflammatory cascade via the competitive utilisation of the NO substrate arginine [20•].

## Dental Caries

The prevalence of dental caries continues to increase worldwide and remains the most common chronic condition during childhood. The assessment of the use of probiotics in the control of dental caries has been limited by the number of subjects needed, prolonged treatment duration and high cost. Most studies have measured counts of *Streptococcus mutans* in saliva or dental plaque; and/or the flow, pH or buffering capacity of saliva. However, only a limited number of studies have used clinical indicators, for both dental caries and the periodontal diseases to demonstrate the efficacy of probiotics. A recent systematic review and meta-analysis provide a useful guide to clinical decision making and direction for further research [31•].

Strains of *Lactobacillus rhamnosus*, *L. casei*, *L. reuteri* and *Bifidobacterium* spp. have all demonstrated the potential to alter colonisation of cariogenic bacteria and thus prevent dental caries [82]. To achieve optimal effects, simultaneous use of multiple species or strains may be required as described in human studies using *Streptococcus oralis*, *S. uberis* and a lactic acid-deficient variant of *S. rattus*, whereby the probiotic mouthwash used was able to markedly affect the levels of cariogenic bacteria in saliva together with periodontal pathogens in subgingival plaque [83, 84]. It is also hypothesised that those oral commensals associated with health are likely to be more effective as probiotic species than the traditional gut-associated probiotic species in terms of ability to colonise, health-promoting functions, biocompatibility and necessary dosage. *Streptococcus dentisani*, a species isolated from individuals who are caries-free, could be a particularly beneficial probiotic species through its production of bacteriocins and acid buffering capacity [35]. As reported in a 90-day clinical study, the probiotic strain *Streptococcus salivarius* M18, also a producer of bacteriocins, was able to reduce caries development in children as assessed by a number of caries-related risk factors [85].

‘Replacement therapy’ based upon biotechnological approaches has also been investigated. Techniques used include gene inactivation to remove harmful metabolites and the incorporation of genes to encode for antimicrobial compounds, for example bacteriocins. *S. mutans* has been considered for replacement therapy in the control of dental caries. A strain of *S. mutans* was made lactate dehydrogenase deficient by the deletion of virtually all of the genetic sequence encoding this enzyme. To compensate for the resulting metabolic imbalance, an alcohol dehydrogenase gene from *Zymomonas mobilis* was then introduced with no detectable lactic acid being produced from the resulting clone. This strain was also significantly less cariogenic than the parent strain as tested in gnotobiotic- and conventional-rodent models of dental caries. In addition, it was found to colonise the teeth of conventional rats to the same extent as the parent strain using both aggressive-displacement and pre-emptive-colonisation approaches. The clone was also shown to be genetically stable and did not revert to producing acid with in vivo and in vitro test systems [86]. It has been suggested that this *S. mutans* clone (SMaRT Replacement Therapy product has recently been developed by Oragenics Inc.) could provide a lifetime of protection in humans against dental caries, but may require occasional re-applications.

## Conclusions

Probiotics could have an important role to play in the clinical management of dental caries and the periodontal diseases, although the evidence is less convincing as regards halitosis. Both long-term efficacy and safety of probiotics should be established in preventative or treatment contexts, in order to inform safe clinical recommendations. Studies must determine strain specific and synergistic effects of strains in vitro to help inform in vivo mechanisms. Innovative approaches using oral microbiome transplants could further increase the role of probiotics in personalised treatment, while other approaches that involve use of more abundant indigenous oral strains may yield long-term benefits in the maintenance of health by the host [87]. Study of the mechanisms involved in the recession of health when oral probiotic loading is concluded may illuminate the role of the host-microbial interface in health.

## References

[1] Rosan B, Lamont RJ (2000). Dental plaque formation. Microbes Infect.

[2] Allaker RP, Douglas CWI (2015). Non-conventional therapeutics for oral infections. Virulence.

[3] Devine DA, Marsh PD (2009). Prospects for the development of probiotics and prebiotics for oral applications. J Oral Microbiol.

[4] Meurman JH (2005). Probiotics: do they have a role in oral medicine and dentistry?. Eur J Oral Sci.

[5] Devine DA, Marsh PD, Meade J (2015). Modulation of host responses by oral commensal bacteria. J Oral Microbiol.

[6] Chapple ILC (2017). Interaction of lifestyle, behaviour or systemic diseases with dental caries and periodontal diseases: consensus report of group 2 of the joint EFP/ORCA workshop on the boundaries between caries and periodontal diseases. J Clin Periodontol.

[7] Hajishengallis G (2015). Periodontitis: from microbial immune subversion to systemic inflammation. Nat Rev Immunol.

[8] Hajishengallis G, Darveau RP, Curtis MA (2012). The keystone pathogen hypothesis. Nat Rev Microbiol.

[9] Nibali L (2015). Aggressive periodontitis: microbes and host response, who to blame?. Virulence.

[10] Saha S (2012). Probiotics as oral health biotherapeutics. Expert Opin Biol Ther.

[11] Teughels W, Loozen G, Quirynen M (2011). Do probiotics offer opportunities to manipulate the periodontal oral microbiota?. J Clin Periodontol.

[12] Montero E et al. Clinical and microbiological effects of the adjunctive use of probiotics in the treatment of gingivitis: a randomized controlled clinical trial. J Clin Periodontol. 2017. 10.1111/jcpe.12752 28556062

[13] Schlagenhauf U (2016). Regular consumption of *Lactobacillus reuteri*-containing lozenges reduces pregnancy gingivitis: an RCT. J Clin Periodontol.

[14] Penala S (2016). Efficacy of local use of probiotics as an adjunct to scaling and root planing in chronic periodontitis and halitosis: a randomized controlled trial. J Res Pharm Pract.

[15] Morales A (2016). Clinical effects of *Lactobacillus rhamnosus* in non-surgical treatment of chronic periodontitis: a randomized placebo-controlled trial with 1-year follow-up. J Periodontol.

[16] Iwasaki K (2016). Daily intake of heat-killed *Lactobacillus plantarum* L-137 decreases the probing depth in patients undergoing supportive periodontal therapy. Oral Health Prev Dent.

[17] Alkaya B, et al. Clinical effects of probiotics containing *Bacillus* species on gingivitis: a pilot randomized controlled trial. J Periodontal Res. 2016:497–504. 10.1111/jre.12415 27859252

[18] Laleman I (2015). The effect of a streptococci containing probiotic in periodontal therapy: a randomized controlled trial. J Clin Periodontol.

[19] Tekce M (2015). Clinical and microbiological effects of probiotic lozenges in the treatment of chronic periodontitis: a 1-year follow-up study. J Clin Periodontol.

[20] Lee J-K (2015). Modulation of the host response by probiotic *Lactobacillus brevis* CD2 in experimental gingivitis. Oral Dis.

[21] Szkaradkiewicz AK, Stopa J, Karpiński TM (2014). Effect of oral administration involving a probiotic strain of *Lactobacillus reuteri* on pro-inflammatory cytokine response in patients with chronic periodontitis. Arch Immunol Ther Exp.

[22] Hallström H (2013). Effect of probiotic lozenges on inflammatory reactions and oral biofilm during experimental gingivitis. Acta Odontol Scand.

[23] Teughels W (2013). Clinical and microbiological effects of *Lactobacillus reuteri* probiotics in the treatment of chronic periodontitis: a randomized placebo-controlled study. J Clin Periodontol.

[24] Vicario M (2013). Clinical changes in periodontal subjects with the probiotic *Lactobacillus reuteri* Prodentis: a preliminary randomized clinical trial. Acta Odontol Scand.

[25] Iniesta M (2012). Probiotic effects of orally administered *Lactobacillus reuteri*-containing tablets on the subgingival and salivary microbiota in patients with gingivitis. a randomized clinical trial. J Clin Periodontol.

[26] Slawik S (2011). Probiotics affect the clinical inflammatory parameters of experimental gingivitis in humans. Eur J Clin Nutr.

[27] Vivekananda MR, Vandana KL, Bhat KG (2010). Effect of the probiotic *Lactobacilli reuteri* (prodentis) in the management of periodontal disease: a preliminary randomized clinical trial. J Oral Microbiol.

[28] Bidault P, Chandad F, Grenier D (2007). Risk of bacterial resistance associated with systemic antibiotic therapy in periodontology. J Can Dent Assoc.

[29] Rams TE, Degener JE, van Winkelhoff AJ (2014). Antibiotic resistance in human chronic periodontitis microbiota. J Periodontol.

[30] Martin-Cabezas R (2016). Clinical efficacy of probiotics as an adjunctive therapy to non-surgical periodontal treatment of chronic periodontitis: a systematic review and meta-analysis. J Clin Periodontol.

[31] Gruner D, Paris S, Schwendicke F (2016). Probiotics for managing caries and periodontitis: systematic review and meta-analysis. J Dent.

[32] Vuotto C, Longo F, Donelli G (2014). Probiotics to counteract biofilm-associated infections: promising and conflicting data. Int J Oral Sci..

[33] Zijnge V (2010). Oral biofilm architecture on natural teeth. PLoS One.

[34] Wade WG (2013). The oral microbiome in health and disease. Pharmacol Res.

[35] Lopez-Lopez A (2017). Health-associated niche inhabitants as oral probiotics: the case of *Streptococcus dentisani*. Front Microbiol.

[36] Toiviainen A (2014). Impact of orally administered lozenges with *Lactobacillus rhamnosus* GG and *Bifidobacterium animalis* subsp. *lactis* BB-12 on the number of salivary mutans streptococci, amount of plaque, gingival inflammation and the oral microbiome in healthy adults. Clin Oral Investig.

[37] • Burton JP et al. Persistence of the Oral probiotic *Streptococcus salivarius* M18 is dose dependent and megaplasmid transfer can augment their bacteriocin production and adhesion characteristics. PLoS One. 2013;8(6). **Study into increasing persistence of the highly abundant probiotic oral streptococcus, used in treating oral malodour and caries**. 10.1371/journal.pone.0065991 PMC3681767 23785463

[38] Vestman NR (2015). Oral microbiota shift after 12-week supplementation with *Lactobacillus reuteri* DSM 17938 and PTA 5289; a randomized control trial. PLoS One.

[39] Dongarrà ML (2013). Mucosal immunology and probiotics. Curr Allergy Asthma Rep.

[40] Ku S (2016). Review on Bifidobacterium bifidum bgn4: functionality and nutraceutical applications as a probiotic microorganism. Int J Mol Sci.

[41] Kobayashi R (2017). Oral administration of *Lactobacillus gasseri* SBT2055 is effective in preventing *Porphyromonas gingivalis*-accelerated periodontal disease. Sci Rep.

[42] McCabe L, Britton RA, Parameswaran N (2015). Prebiotic and probiotic regulation of bone health: role of the intestine and its microbiome. Curr Osteoporos Rep.

[43] Pazzini CA (2017). Probiotic consumption decreases the number of osteoclasts during orthodontic movement in mice. Arch Oral Biol.

[44] İnce G (2015). Clinical and biochemical evaluation of *Lactobacillus reuteri* containing lozenges as an adjunct to non-surgical periodontal therapy in chronic periodontitis. J Periodontol.

[45] Oliveira LFF (2017). Benefits of *Bifidobacterium animalis* subsp *lactis* probiotic in experimental periodontitis. J Periodontol.

[46] Messora MR (2016). Favourable effects of *Bacillus subtilis* and *Bacillus licheniformis* on experimental periodontitis in rats. Arch Oral Biol.

[47] Foureaux RDC (2014). Effects of probiotic therapy on metabolic and inflammatory parameters of rats with ligature-induced periodontitis associated with restraint stress. J Periodontol.

[48] Maekawa T, Hajishengallis G (2014). Topical treatment with probiotic *Lactobacillus brevis* CD2 inhibits experimental periodontal inflammation and bone loss. J Periodontal Res.

[49] Mendi A (2016). *Lactobacillus rhamnosus* could inhibit *Porphyromonas gingivalis* derived CXCL8 attenuation. J Appl Oral Sci.

[50] Bene KP (2017). *Lactobacillus reuteri* surface mucus adhesins upregulate inflammatory responses through interactions with innate C-Type lectin receptors. Front Microbiol.

[51] Godoy L, et al. The probiotic mixture VSL#3 alters the morphology and secretion profile of both polarized and unpolarised human macrophages in a polarization-dependent manner. J Clin Cell Immunol. 2014;5(3) 10.4172/2155-9899.1000227 PMC4145411 25177525

[52] Scully C, Greenman J (2008). Halitosis (breath odor). Periodontol.

[53] Bollen CM, Beikler T (2012). Halitosis: the multidisciplinary approach. Int J Oral Sci.

[54] Allaker RP (2008). Topographic distribution of bacteria associated with oral malodour on the tongue. Arch Oral Biol.

[55] Zaura E (2009). Defining the healthy “core microbiome” of oral microbial communities. BMC Microbiol.

[56] Eren a M (2014). Oligotyping analysis of the human oral microbiome. Proc Natl Acad Sci.

[57] Wescombe PA (2010). Something old and something new: an update on the amazing repertoire of bacteriocins produced by *Streptococcus salivarius*. Probiotics Antimicrob Proteins.

[58] Horz HP (2007). Distribution and persistence of probiotic *Streptococcus salivarius* K12 in the human oral cavity as determined by real-time quantitative polymerase chain reaction. Oral Microbiol Immunol.

[59] Burton JP (2006). A preliminary study of the effect of probiotic *Streptococcus salivarius* K12 on oral malodour parameters. J Appl Microbiol.

[60] Masdea L (2012). Antimicrobial activity of *Streptococcus salivarius* K12 on bacteria involved in oral malodour. Arch Oral Biol.

[61] Iwamoto T (2010). Effects of probiotic *Lactobacillus salivarius* WB21 on halitosis and oral health: An open-label pilot trial. Oral Surgery, Oral Med Oral Pathol Oral Radiol. Endodontology.

[62] Suzuki N (2014). *Lactobacillus salivarius* WB21-containing tablets for the treatment of oral malodor: a double-blind, randomized, placebo-controlled crossover trial. Oral Surg Oral Med Oral Pathol Oral Radiol.

[63] Jang HJ, et al. Comparative study on the characteristics of *Weissella cibaria* CMU and probiotic strains for oral care. Molecules. 2016;21(12) 10.3390/molecules21121752 PMC6274271 27999400

[64] Suzuki N et al. Inhibitory effect of enterococcus faecium wb2000 on volatile sulfur compound production by porphyromonas gingivalis. Int J Dent. 2016. 10.1155/2016/8241681 PMC5075313 27799940

[65] Lee S-H, Baek D-H (2014). Effects of *Streptococcus thermophilus* on volatile sulfur compounds produced by *Porphyromonas gingivalis*. Arch Oral Biol.

[66] Fujiwara N (2017). Novel reuterin-related compounds suppress odour by periodontopathic bacteria. Oral Dis.

[67] Keller MK (2012). Effect of chewing gums containing the probiotic bacterium *Lactobacillus reuteri* on oral malodour. Acta Odontol Scan.

[68] Marchetti E (2015). Multi-sensor approach for the monitoring of halitosis treatment via *Lactobacillus brevis* (CD2)-containing lozenges—a randomized, double-blind placebo-controlled clinical trial. Sensors (Basel).

[69] Sutula J (2013). The effect of a commercial probiotic drink containing *Lactobacillus casei* strain Shirota on oral health in healthy dentate people. Microb Ecol Health Dis.

[70] Terai T (2015). Screening of probiotic candidates in human oral bacteria for the prevention of dental disease. PLoS One.

[71] Mancinelli RL, McKay CP (1983). Effects of nitric oxide and nitrogen dioxide on bacterial growth. Appl Environ Microbiol.

[72] Kilian M (2016). The oral microbiome—an update for oral healthcare professionals. Brit Dent J.

[73] Lundberg JO (2004). Nitrate, bacteria and human health. Nat Rev (Microbiol).

[74] Doel JJ (2005). Evaluation of bacterial nitrate reduction in the human oral cavity. Eur J Oral Sci.

[75] Benjamin N (1994). Stomach NO synthesis. Nat.

[76] Silva Mendez LS (1999). Antimicrobial effect of acidified nitrite on cariogenic bacteria. Oral Microbiol Immunol.

[77] Doel JJ (2004). Protective effect of salivary nitrate and microbial nitrate reductase activity against caries. Euro. J Oral Sci.

[78] Stephen AS (2017). The role of periodontal niches in influencing the tongue microbiota: relationship with periodontitis and oral malodour. J Oral Microbiol.

[79] Qu XM (2016). From nitrate to nitric oxide: the role of salivary glands and oral bacteria. J Dent Res.

[80] Hussain QA (2016). Detection of adrenomedullin and nitric oxide in different forms of periodontal disease. J Perio Res.

[81] Hussain QA (2015). Regulation of adrenomedullin and nitric oxide production by periodontal bacteria. J Perio Res.

[82] Meurman JH, Stamatova I (2007). Probiotics: contributions to oral health. Oral Dis.

[83] Haukioja A (2010). Probiotics and oral health. Euro. J Dent.

[84] Zahradnik RT (2009). Preliminary assessment of safety and effectiveness in humans of ProBiora3, a probiotic mouthwash. J Appl Microbiol.

[85] Pierro D (2015). Cariogram outcome after 90 days of oral treatment with *Streptococcus salivarius* M18 in children at high risk for dental caries: results of a randomized, controlled study. Clin Cosmet Invest Dent.

[86] Hillman JD (2000). Construction and characterization of an effector strain of *Streptococcus mutans* for replacement therapy of dental caries. Infect Immun.

[87] Pozhitkov AE (2015). Towards microbiome transplant as a therapy for periodontitis: an exploratory study of periodontitis microbial signature contrasted by oral health, caries and edentulism. BMC Oral Health.

